# Dietary Intake and Its Association with Birth Outcomes in Women with Nausea and Vomiting during the Second Trimester of Pregnancy: A Prospective Cohort Study in Japan

**DOI:** 10.3390/nu15153383

**Published:** 2023-07-29

**Authors:** Nao Nishihara, Megumi Haruna, Yuriko Usui, Kaori Yonezawa, Naoko Hikita, Emi Sasagawa, Keiko Nakano, Moeko Tanaka, Riko Ohori, Satoko Aoyama, Satoshi Sasaki, Megumi Fujita, Masayo Matsuzaki, Yoshiko Suetsugu, Yoko Sato

**Affiliations:** 1 Department of Midwifery and Women’s Health, Division of Health Sciences and Nursing, Graduate School of Medicine, The University of Tokyo, Tokyo 113-0033, Japan; nishihara-nao185@g.ecc.u-tokyo.ac.jp (N.N.); yusui@g.ecc.u-tokyo.ac.jp (Y.U.); kaoriyone@m.u-tokyo.ac.jp (K.Y.); hikita.naoko.419@m.kyushu-u.ac.jp (N.H.); e-sasagawa@redcross.ac.jp (E.S.); nknkco3o3o@gmail.com (K.N.); tanaka-moeko48526@g.ecc.u-tokyo.ac.jp (M.T.); r.i_11_k.o_08@icloud.com (R.O.); aoyama-satoko125@g.ecc.u-tokyo.ac.jp (S.A.); 2 Global Nursing Research Center, Graduate School of Medicine, The University of Tokyo, Tokyo 113-0033, Japan; 3 Department of Health Sciences, Graduate School of Medical Sciences, Kyushu University, Fukuoka 812-8582, Japan; suetsugu.yoshiko.742@m.kyushu-u.ac.jp (Y.S.); satou.youko.350@m.kyushu-u.ac.jp (Y.S.); 4 Department of International Health Care and Midwifery, Graduate School of Nursing, Japanese Red Cross College of Nursing, Tokyo 150-0012, Japan; 5 Department of Social and Preventive Epidemiology, School of Public Health, The University of Tokyo, Tokyo 113-0033, Japan; stssasak@m.u-tokyo.ac.jp; 6 Department of Clinical Nursing, Graduate School of Medical Science, Yamagata University, Yamagata 990-9585, Japan; f.megumi@med.id.yamagata-u.ac.jp; 7 Department of Reproductive Health Nursing, Graduate School of Health Care Sciences, Tokyo Medical and Dental University, Tokyo 113-8510, Japan; matsu3@sahs.med.osaka-u.ac.jp; 8 Department of Children and Women’s Health, Division of Health Sciences, Graduate School of Medicine, Osaka University, Osaka 565-0871, Japan

**Keywords:** pregnancy, nausea, vomiting, PUQE, diet, GWG

## Abstract

Nausea and vomiting in pregnancy (NVP) is a common symptom. Although the influence of NVP during the first trimester on dietary intake and birth outcomes has been revealed, no study has focused on NVP during the second trimester. This study aimed to reveal whether NVP severity during the second trimester is associated with dietary intake, gestational weight gain (GWG), birth weight, and delivery week. Participants completed a questionnaire at 18–27 gestational weeks. NVP severity was assessed using the modified Pregnancy-Unique Quantification of Emesis and Nausea scale in the questionnaire. Dietary habits were assessed using a brief-type diet history questionnaire. In total, 825 responses were analyzed: 202 (24.5%), 135 (16.4%), and 8 (1.0%) women reported mild, moderate, and severe NVP, respectively; 480 (58.2%) women did not have NVP during the second trimester. No significant association was observed between energy and nutrient intake and no/mild and moderate/severe NVP. Women with moderate/severe NVP had lower total GWG than those with no/mild NVP (*p* = 0.007). There was no significant difference in low birth weight and preterm birth rates (*p* = 0.246 and *p* = 0.604). This is the first study to investigate whether NVP severity during the second trimester is associated with dietary intake and birth outcomes.

## 1. Introduction

Nausea and vomiting in pregnancy (NVP) is one of the most common symptoms among pregnant women and is prevalent in 70–80% of them [1]. Although the pathogenesis of NVP remains unclear, previous studies have shown that various factors are involved in NVP: reproductive hormones (human chorionic gonadotropin, hCG; estrogen and progesterone), helicobacter pylori, and gastric slow-wave rhythm disorder [1,2]. NVP begins at 8 weeks and peaks at 11–13 gestational weeks [1,3]. Many studies on NVP in the first trimester have been conducted; however, only a few studies have focused on NVP during the second trimester. Although more than 40% of pregnant women experience NVP during the second trimester, more than 35% of them experience moderate or severe NVP [4,5]. Severe NVP is associated with hospitalization for NVP during the first trimester [6].

Nutritional intake during pregnancy is important for both maternal and infant health. During pregnancy, the required amounts of energy and nutrients increase; for pregnant Japanese women, protein, magnesium, iron, zinc, copper, vitamin B1, vitamin B2, vitamin B6, vitamin B12, folate, and vitamin C are the nutrients with the estimated average requirements [7]. However, a previous study in Japan showed that the dietary intake of energy, protein, and some micronutrients was insufficient during the second trimester [8]. Few studies have been conducted on women with NVP and their dietary intake. Several studies have revealed that women with severe NVP in the first trimester had a lower intake of energy, macronutrients, and micronutrients than those with less severe NVP or without NVP [9,10,11], while a cohort study conducted in Norway showed inconsistent results, in which women with NVP had a higher energy intake than those without [12]. However, no studies have been conducted to determine the association between NVP during the second trimester and dietary intake as yet.

The association between small GWG in pregnant women and severe NVP or vomiting during the first trimester has been previously revealed [11,12]. Inadequate GWG is associated with adverse birth outcomes; thus, attaining adequate GWG is important in pregnant women [13,14]. Although a negative effect of NVP during the first trimester on dietary intake and GWG has been reported, a previous study showed that women with NVP during the first or early pregnancy (first to second trimester) had a lower risk of preterm birth and low birth weight [15,16]. However, no study has revealed whether NVP during the second trimester is associated with GWG, infant birth weight, or delivery week.

Therefore, this study aimed to determine whether NVP severity during the second trimester is associated with dietary intake, GWG, birth weight, and preterm birth.

## 2. Materials and Methods

This study was conducted as part of the Japan Pregnancy Eating and Activity Cohort (J-PEACH) Study, a prospective cohort study conducted in four cities in Japan: Yamagata, Tokyo, Osaka, and Fukuoka [17]. The population of Yamagata is smaller than the other three cities; approximately 0.2 million for Yamagata, 9.5 million for Tokyo, 2.7 million for Osaka, and 1.5 million for Fukuoka [18]. Participants were recruited during their prenatal care visits between March 2020 and October 2022. However, the recruitment part was suspended because of the Coronavirus disease 2019 (COVID-19) pandemic. Inclusion criteria were age ≥18 years, ability to read and write in Japanese, and expectation of delivery at the study participants’ hospital. Exclusion criteria were medical staff-deemed inability to participate and hospitalization for the treatment of hyperemesis gravidarum (HG). HG is defined as an extreme form of NVP, associated with weight loss, dehydration, and electrolyte deficiency [19]. HG prevalence is 1.2–1.6%, and HG is associated with hospitalization [20,21]. The enrolled participants received a questionnaire after 18 gestational weeks and completed it at 18–27 gestational weeks.

**Figure 1:**
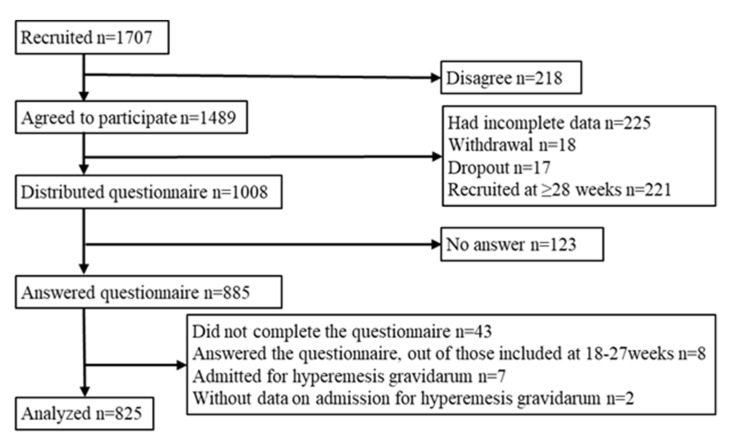
Flowchart of the participants.

In the J-PEACH Study, 1707 pregnant women were recruited, and 1489 (87.2%) agreed to participate. After excluding those with incomplete data (n = 225), withdrew (n = 18), dropped out (n = 17), and had a gestational age >28 weeks (n = 221), the questionnaires were distributed to 1008 women in their second trimester (Figure 1). In total, 885 participants answered, while 842 participants completed the questionnaire. The response rate was 82.5%.

Participants who answered the questionnaire out of those with a gestational age of 18–27 weeks (n = 8), who were admitted for HG (n = 7), and without data on admission for HG (n = 2) were excluded. Finally, 825 women were included in this analysis.

The participants received the survey link via email. The questionnaire included questions regarding NVP symptoms, demographic information, psychological status, and dietary intake.

Participants were asked about their NVP experience in the past month (yes/no). Only women with NVP were subjected to the modified Pregnancy-Unique Quantification of Emesis and Nausea (PUQE) Scale to assess NVP severity. The original PUQE focused on NVP symptoms in the last 12 h [22], and it was modified to feature symptoms experienced over a wider period of time [5]. In this study, the modified PUQE was used because the recall period was equal to that of the dietary intake questionnaire. The modified PUQE was validated in English, and the validated Japanese version of the PUQE-24 was used, excluding period [23]. The modified PUQE uses a five-point Likert scale and is based on three symptoms of NVP: hours of nausea experience, number of retching episode, and number of vomiting episode on an average day in the past month. The total score ranged from 3 to 15, and was used to classify NVP as mild (3–6), moderate (7–12), or severe (≥13) [24]. Participants were then divided into two groups: no/mild NVP and moderate/severe NVP.

The questionnaire was also used to obtain information on demographic factors such as marital status, educational level, annual household income, and smoking status. Educational level was categorized as junior high school or high school, vocational training school or junior college, college, and postgraduate. Annual household income (Japanese yen) was divided into <7 million and ≥7 million. A household income of JPY 7 million was almost equal to the average household income of families with children in Japan [25]. Smoking status during pregnancy was considered a dichotomized variable (yes/no).

Information on the expected date of delivery (EDD), age, parity, and admission due to HG was obtained from the medical records. The gestational week at the time of answering the questionnaire was calculated using the EDD and response date. Self-reported weight and height were also obtained from the medical records. Pre-pregnancy BMI was calculated and categorized as <18.5 (underweight), 18.5–24.9 (normal weight), 25.0–29.9 (overweight), and ≥30.0 kg/m^2^ (obese) [26].

Dietary habits during the preceding month were assessed using a brief-type diet history questionnaire (BDHQ), which queried the consumption frequency of selected food and beverage items [27,28]. The intake of energy and 58 nutrients was estimated using an ad hoc computer algorithm (including weighting factors). The BDHQ has previously been validated in pregnant Japanese women [29]. Information regarding the number of consumed meals and skipped meals was collected using the questionnaire. Number of meals was defined as ≥50 kcal, with an interval of ≤15 min, during weekdays in the past month. Regarding skipped meals, participants were asked, “On weekdays over the last 1 month, which meal did you mainly skip?” and they answered, “breakfast”, “lunch”, “dinner”, or “mostly did not skip breakfast”. Participants with NVP answered a question about their degree of dietary intake and were categorized as experienced NVP and ate more than usual, ate as usual, ate less than usual, or could hardly eat. In addition, regarding the question about who prepares the meals, participants answered “self” or “others”. Regarding the question about food nutrition label checks for nutrients and calories, they answered, “yes” or “no”.

The GWG at each trimester and total GWG were calculated by subtracting the self-reported pre-pregnancy weight from the weight obtained from the medical records. Weight measured at antenatal visits and their corresponding gestational weeks were collected from medical records, and the week closest to the gestational weeks for each trimester was extracted for each woman: 12 weeks for the first trimester and 24 weeks for the second trimester. Pre-delivery weight, defined as the last weight measured before delivery, was also obtained from the medical records. The GWG increase rate between each trimester was calculated by dividing the weight difference by the gestational week difference. The total GWG increase rate was also calculated. Women with a total GWG lower than the minimum recommended value were defined as having insufficient GWG [26].

Information regarding birth weight and delivery week was also obtained from the medical records. Low birth weight and preterm birth were defined as birth weights of <2500 g and delivery at <37 weeks, respectively.

Women who did not complete the study questionnaire were excluded from the analysis. The Shapiro–Wilk test was conducted to examine the normality of the distribution of the variables. Comparisons of the demographic and psychological variables between the no/mild and moderate/severe NVP groups were evaluated using the Pearson’s chi-square test for categorical variables and the Mann–Whitney U test for continuous variables as none of the continuous variables were normally distributed. The no/mild and moderate/severe NVP groups for the total GWG, GWG at each trimester, and birth weight were compared using the Mann–Whitney U test and divided into pre-pregnancy BMI categories. The GWG increase rate and delivery week were compared using the Mann–Whitney U test. Participants with multiple pregnancies, preterm births, and a lack of data on delivery week were excluded from the GWG analysis. For birth weight and delivery week, only those who had multiple births and with a lack of data were excluded.

Participants that reported extremely unrealistic energy intakes were excluded from the analysis. Specifically, a reported energy intake less than half of the energy requirement for the lowest physical activity category or intake that was 1.5 times higher than the energy requirement for the moderate physical activity category was excluded [30]. Dietary intake between the two NVP groups was compared using the Mann–Whitney U test. Nutrients with estimated average requirements for pregnant Japanese women were included in the analysis [7]. Statistical significance was set at *p* < 0.05. All analyses were performed using SPSS software version 29.0 for Windows (IBM Corp., Armonk, NY, USA).

The sample size was calculated using G*Power [31]. The amount of energy consumed was indicated as an outcome. Based on previous research, the effect size was determined to be 0.257 and the sample size was calculated at the 5% level with a power of 80% and an allocation ratio of 0.211 [5,32]. The estimated sample size was 868.

## 3. Results

### 3.1. Nausea and Vomiting in Pregnancy

In total, 480 (58.2%) women did not have NVP, and 202 (24.5%), 135 (16.4%), and eight (1.0%) reported mild, moderate, and severe NVP, respectively, during the second trimester. Among the women with mild NVP, only 37 (18.3%) experienced vomiting at a frequency of 1–2 times a day. On the other hand, among those with moderate NVP, 76 (56.3%) experienced vomiting and 12 (8.9%) experienced vomiting more than three times a day. All women with severe NVP experienced vomiting more than three times a day.

### 3.2. Demographic Characteristics

For the analysis, the participants were divided into two groups: no/mild NVP (n = 682) and moderate/severe NVP (n = 143). The median gestational week (25th–75th quartile [Q1–Q3]) was 22.0 (19.0–24.0), the median age of the participants was 34.0 (31.0–38.0) years, 393 (47.6%) women were multiparous, 601 (73.1%) women had normal BMI, and 15 (1.8%) were not married. Regarding education, 445 (53.9%) and 73 (8.8%) had college and postgraduate education, respectively, and 533 (64.6%) were employed (Table 1).

Women with moderate/severe NVP were significantly more likely to have a lower gestational age, were unmarried, and were less likely to smoke compared with those with no/mild NVP. No significant differences were observed in parity, number of fetuses, pre-pregnancy BMI, education, work, household income, or city.

As for the cities, the participants from Yamagata were significantly less educated (college or postgraduate: 43.4%, *p* < 0.001) and had less household income (≥7 million yen: 39.6%, *p* < 0.001) than those from the other three cities; Tokyo, Osaka, and Fukuoka. No significant difference was observed in age, parity, the number of fetuses, marital status, and pre-pregnancy BMI between Yamagata and the other three cities.

### 3.3. Dietary Intake

The energy intakes, body-mass-adjusted intakes of macronutrients and energy-adjusted micronutrients of women with no/mild NVP and those with moderate/severe NVP are compared in Table 2. Those with unrealistic energy intakes were excluded from the analysis of the BDHQ: one (0.1%) for over-reporting in the no/mild NVP group and 47 (6.9%) and 22 (15.4%) for under-reporting in the no/mild and moderate/severe NVP groups, respectively. In total, the BDHQ data from 755 women were analyzed. The excluded 70 participants were significantly more likely to be either primipara (n = 46, 65.7%) or unmarried (n = 4, 5.7%) than the 755 analyzed women.

There were no significant differences in the energy intake, body-mass-adjusted intakes of macronutrients and energy-adjusted micronutrients between the two NVP groups. Regarding the degree of dietary intake, of the 365 women with NVP, 36 (4.4%) answered that they ate more than usual, 198 (24.0%) ate as usual, 102 (12.4%) ate less than usual, and nine (1.1%) could hardly eat.

A comparison of the effect of energy and macronutrient and micronutrient intakes on the degree of dietary intake was conducted; women who ate more than usual had significantly higher energy and nutrient intakes than those without NVP. However, when women who ate more than usual were excluded, the results of the comparison of the energy and nutritional intakes between women with no/mild NVP and those with moderate/severe NVP did not differ.

Women in the moderate/severe NVP group were more likely to consume noodles, pickled vegetables, tea, and juice than those in the no/mild NVP group (Table 3).

The dietary habits of women with no/mild NVP and those with moderate/severe NVP are compared in Table 4. Women with moderate/severe NVP were significantly more likely to skip meals than those with no/mild NVP during the second trimester (*p* = 0.003). Skipped meals on weekdays included breakfast (137; 68.8%), lunch (27; 13.6%), dinner (25; 12.6%), breakfast and lunch (4; 2.0%), breakfast and dinner (4; 2.0%), and all three meals (2; 1.0%). In total, 37 (25.9%) women with moderate/severe NVP skipped breakfast, a percentage significantly higher than that of women with no/mild NVP (110, 16.1%). Number of meals including snack was not significantly different between the two groups, as both had a median (Q1–Q3) of 4.0 meals (3.0–4.0) a day. The moderate/severe NVP group was significantly less likely to check food nutrition labels for nutrients than the no/mild NVP group, whereas checks for calories was not significantly different. More than 90% of the participants prepared their meals, with no significant difference between the two NVP groups.

### 3.4. Gestational Weight Gain, Low Birth Weight, and Preterm Birth

Table 5 shows the results of the comparison of GWG, birth weight, and delivery week between women with no/mild and those with moderate/severe NVP. The total GWG, first trimester GWG, and second trimester GWG were 9.9, 0.3, and 4.1 kg, respectively.

Regarding GWG, women who had multiple pregnancies or preterm births were excluded, and data from 684 women were analyzed. Women with moderate/severe NVP had a smaller total GWG (median, [Q1–Q3]) than those with no/mild NVP (10.0 (7.9–12.1) vs. 9.0 (6.4–11.4) kg). Regarding the pre-pregnancy BMI category, no significant difference remained, but for the pre-pregnancy BMI 18.5–24.9 kg/m^2^ (normal weight) group, women with moderate/severe NVP seemed to have a smaller total GWG (*p* = 0.050). Regarding GWG in the first and second trimesters, both the overall participant population and pre-pregnancy BMI 18.5–24.9 kg/m^2^ group comparisons showed smaller GWG for women with moderate/severe NVP than for those with no/mild NVP. For all participants, the proportion of patients with insufficient GWG was 51.5%, which was not significantly different between the two groups. Regarding the GWG increase rate, the moderate/severe NVP group had significantly smaller rates than the no/mild group during the first trimester, around 12 weeks (−0.02 [−0.15–0.08] vs. 0.03 [−0.06–0.13] kg). Significance was only observed in the pre-pregnancy BMI 18.5–24.9 group (normal weight). However, no significant difference was observed in the GWG increase rate during the second trimester.

Regarding birth weight and delivery week, only women with multiple pregnancies were excluded, and data from 743 and 736 women, respectively, were analyzed. Median (Q1–Q3) birth weight and delivery week were 3020.0 g (2752.0–3288.0) and 39.0 (38.0–40.0), respectively. The rates of low birth weight and preterm births were 7.0% and 8.1%, respectively, with no significant differences between the two groups.

## 4. Discussion

This study was part of the J-PEACH Study, a prospective cohort study conducted in four cities in Japan. The present study examined whether NVP severity during the second trimester was associated with dietary intake, GWG, birth weight, and preterm birth. No significant association was observed between energy, macronutrient, and micronutrient intakes and no/mild and moderate/severe NVP. Women with moderate/severe NVP had a smaller total GWG than those with no/mild NVP, with no significant differences in birth weight, delivery week, and rates of low birth weight and preterm birth.

The prevalence rates of mild, moderate, and severe NVP were 24.5%, 16.4%, and 1.0%, respectively, implying that more than 40% experienced NVP and 17.4% experienced moderate or severe NVP during the second trimester. This was almost consistent with the result of a previous study in Canada that measured the NVP severity during the second trimester using a modified PUQE scale (25.4% for mild, 14.4% for moderate, and 0.3% for severe) [5]. Women with moderate/severe NVP during the second trimester had a significantly earlier gestational age than those with no/mild NVP in the present study. Although a previous study showed that the severity of nausea and retching decreased as gestational age increased during the first trimester [33], this is the first study to reveal that the severity of NVP during the second trimester also decreases as gestational age increases.

In the current study, there was no significant difference in energy, macronutrient, and micronutrient intakes between women with moderate/severe NVP and those with no/mild NVP during the second trimester. This finding was inconsistent with those reported in previous studies showing that women with severe NVP in the first trimester had lower intakes of energy, macronutrients, and micronutrients than those with less severe NVP or without NVP [9,10,11]. Regarding meal skipping in the present study, women in the moderate/severe NVP group were more likely to skip meals, especially breakfast, but the number of meals including snacks was not significantly different from that reported in the no/mild group. This implies that women with moderate/severe NVP may be able to complement their dietary intake by consuming frequent meals including snacks, even though they are more likely to skip meals.

However, it is necessary to consider that the BDHQ has low validity for estimating energy intake in women with NVP. In a previous study on BDHQ validation among pregnant women, the estimated energy from the BDHQ was higher than that from the 3-day dietary record only among women with NVP [29]. Energy intake is more likely to be overestimated in the moderate/severe NVP group than in the no/mild NVP group. Additionally, as women with NVP are more likely to underreport their dietary intake [34], the exclusion rate, the rate of answering the unrealistic energy intake of BDHQ was higher in the moderate/severe NVP group than in the no/mild group (15.4% vs. 6.9%). This measurement limitation may have a greater effect on the moderate/severe NVP group than on the no/mild NVP group in terms of statistical indicators of energy intake.

Women in the moderate/severe NVP group were more likely to consume noodles, pickled vegetables, tea, and juice than those in the no/mild NVP group in this study. Women with moderate/severe NVP were also significantly less likely to check food nutrition labels for nutrients than those with no/mild NVP, whereas the likelihood of checking calories was not significantly different between the groups. A previous review showed that women with NVP tended to exhibit food aversions and cravings [35]. In the present study, cravings for noodles and juice were consistent with the results of previous studies, whereas aversions to certain foods such as eggs, tea, some meat, and vegetables were not observed in other studies [11,12,35]. This inconsistency may have occurred due to differences in NVP period or country. Furthermore, >90% of the participants prepared meals themselves, and women with moderate/severe NVP had food cravings. Therefore, they may not be able to afford to choose their meals based on nutrition labels and may prefer foods that are easier to cook such as noodles. In Japan, a variety of frozen foods and pre-packed foods are sold. They also contain various ingredients such as vegetables; therefore, women with NVP may be able to have vegetables. Moreover, pickled vegetables in Japan taste salty, not sour. Hormonal changes during pregnancy have an impact on taste function, less salty intensity, while sour and sweet does not change [36]. This difference may be a reason for vegetable intake.

This study is the first to evaluate differences in dietary intake focusing on NVP during the second trimester. Although a relative comparison of nutritional intake levels may have been conducted, the absolute amount of nutrient intake remains unclear. Clinical staff should apply caution regarding sufficient nutrient intake during pregnancy, as pregnant women require more amounts of certain nutrients than they did before pregnancy [7].

For all participants in our study, the total GWG, first-trimester GWG, and second-trimester GWG were 9.9, 0.3, and 4.1 kg, respectively. Compared with the participants in a previous large cohort study in Japan, in our study, the proportion of the participants in the pre-pregnancy BMI 18.5–24.9 kg/m^2^ (normal weight) group were almost similar; however, they were slightly lower in the <18.5 (underweight) group and higher in the 25.0–29.9 (overweight) and ≥30.0 kg/m^2^ (obese) groups. Categorizing the participants into pre-pregnancy BMI groups ensured that the impact of pre-pregnancy BMI was adjusted for. The total, first, and second trimester GWG were slightly smaller in our study than those reported in previous studies, except in the pre-pregnant BMI ≥30.0 kg/m^2^ (obese) group [37]. This may have occurred because of the higher age of the participants of this study than that of the participants of previous studies. The higher the age, the smaller the GWG, except in the pre-pregnant BMI ≥ 30.0 kg/m^2^ (obese) group [37].

Women with moderate/severe NVP had a smaller total GWG than those with no/mild NVP, and only women in the pre-pregnancy BMI 18.5–24.9 kg/m^2^ (normal weight) group had significantly smaller GWG in the first and second trimesters. A previous study in Norway showed that vomiting during early pregnancy and pre-pregnancy BMI ≥ 18.5 kg/m^2^ was associated with a smaller total GWG, whereas no significance was observed for pre-pregnancy BMI <18.5 kg/m^2^ (underweight) [12]. Another study conducted in China revealed an association between a lower GWG and severe NVP during the first trimester [11]. Regarding the pre-pregnancy BMI <18.5 (underweight) and 18.5–24.9 kg/m^2^ (normal weight) groups, the results were consistent with those of previous studies, while inconsistencies were observed for the 25.0–29.9 (overweight) and ≥30.0 kg/m^2^ (obese) groups, possibly due to the small sample size in the present study compared with those in previous studies.

The GWG increase rate was significantly smaller in the moderate/severe NVP group than in the no/mild NVP group during the first trimester, around 12 weeks. Significance was only observed in the pre-pregnancy BMI 18.5–24.9 (normal weight) group. As NVP peaks during the first trimester and improves as gestational age progresses [1], women with moderate/severe NVP during the second trimester may experience more severe NVP than those with no/mild NVP during the second trimester. Although NVP severity during the first trimester was not observed in this study, the association between the smaller GWG in the first trimester and moderate/severe NVP in the second trimester may be explained. There was no significant difference in the rate of GWG increase in the second trimester between the NVP groups. This implies that, even though women who experienced moderate/severe NVP during the second trimester had a lower rate of GWG increase in the first trimester, they could catch up from the second trimester. However, because of the lower rate of GWG increase in the first trimester, the total GWG was slightly lower in the moderate/severe NVP group than in the no/mild NVP group. The impact seemed relatively small, so the proportion of patients with insufficient GWG was not significantly different between the two groups.

In this study, for all the participants, the median (Q1–Q3) birth weight and delivery week were 3020.0 (2752.0–3288.0) g, and 39.0 (38.0–40.0), respectively. The rates of low birth weight and preterm birth were 7.0% and 8.1%, respectively. Although birth weight and delivery week were almost similar to those reported in a previous study and a survey conducted in Japan, the rate of low birth weight was lower and that of preterm birth was higher (8.1% and 4.6–4.7%, respectively) in our study [16,38]. This inconsistency may be because our study was conducted at university hospitals and the patients were more likely to be at high risk. Additionally, compared with the previous large cohort study in Japan, the proportion of the pre-pregnancy BMI was slightly lower for the BMI <18.5 (underweight) group, and higher for the 25.0–29.9 (overweight) and ≥30.0 kg/m^2^ (obese) groups. The previous study showed that women with a pre-pregnancy BMI <18.5 have higher odds of the incidence of low birth weight than those with 25.0–29.9 and ≥30.0 kg/m^2^ [39].

Inconsistent with a previous study, no significant association was found between moderate/severe NVP during pregnancy and low birth weight or preterm birth in this study. Regarding low birth weight, a large cohort study conducted in Norway showed that women who experienced only nausea during early pregnancy had a lower risk of low birth weight than women without symptoms [15]. A large cohort study conducted in Japan revealed that women who experienced only nausea in the first 12 weeks had a lower risk of preterm birth than those who did not experience NVP [16]. These inconsistencies may have occurred because of the differences in NVP category, the presence of NVP symptoms only [15], and the presence of NVP symptoms and whether they could eat [16]. However, the frequency of these symptoms varies among pregnant women [40]. Additionally, to consider low birth weight and preterm birth as outcomes, a study with a larger sample size is required.

This study had several limitations. First, selection bias was presumed to occur. Specifically, women with severe NVP or poor mental health were less likely to answer the questionnaire. To minimize bias, the questionnaire was divided into two parts to reduce the burden on the participants; thus, the current study had a high response rate. Second, the study was conducted during the COVID-19 infection period, which has had an enormous impact on lifestyle. As the participants possibly had limitations in going out, their manner of eating varied dramatically. Moreover, people are becoming more conscious of their health. Although this study may have been affected by COVID-19, the data have valuable implications for after-COVID-19 lifestyle.

As for the background of the participants, they had a higher median age and higher education than the survey conducted in Japan [41,42] whereas the household income and the proportion of primipara and pre-pregnancy BMI were almost consistent [25,41,43]. This may have occurred because the study was mainly conducted at university hospitals in the urban area and the participants were more likely to be at high risk. This suggests that the participants were more conscious of their health, which may have led to efficient dietary intake, even in participants with moderate/severe NVP. Therefore, the results of this study should be interpreted with caution. Although the population of Yamagata is smaller than the other three cities, and the participants from Yamagata were less educated and had less household income than those from the other three cities, the inclusion of Yamagata may slightly improve the representativeness of the current study.

In this study, NVP severity was only assessed by the self-reported questionnaire and objective measurements were missing. This is one of the limitations of this study. However, the severity of NVP is associated with the quality of life of pregnant women; focusing on NVP not as severe as hospitalization for HG was necessary [24]. As for the other possible causes of nausea and vomiting, none of the participants had cholecystitis liver, biliary tract diseases, and drug abuse according to the medical records. In this study, the information for gastroenteritis could not be obtained, which is one of the limitations. However, focusing on NVP during the past month may minimize the effect.

Moreover, information on antiemetics use could not be obtained in this study. The use of antiemetics may relieve NVP severity, and the participants using antiemetics can be categorized as the less severe NVP group than those who did not use antiemetics. The participants using antiemetics may experience more severe NVP when the effect of the medicine wears off. However, regardless of the antiemetics use, NVP severity, measured by the frequency of the symptoms experienced by the participants during the past month, was the focus of this study.

Regarding the BDHQ, energy intake measured using this tool has been reported to have low validity, and the dietary intake of women with NVP is likely to be overestimated [29,34]. The energy-adjusted intakes of macronutrients, micronutrients, and food groups, which were validated in a previous study, was used to minimize this influence. In addition, some of the questions included in the questionnaire were about past events; thus, recall bias is another limitation.

Although this study had some limitations, it is the first to reveal whether NVP severity during the second trimester is associated with dietary intake, lower GWG, low birth weight, and preterm birth. The study was conducted in four regions in Japan; thus, it has high generalizability.

## 5. Conclusions

The proportion of women with NVP during the second semester was more than 40% in this study, while the proportion of those with severe NVP was 17.4%. Although no differences in energy-adjusted macronutrient and micronutrient intakes were observed, the clinical staff should still apply caution regarding sufficient nutrient intake during pregnancy based on the dietary intake of women with NVP during the second trimester. Further research is required regarding the effects of NVP on mental health during the second trimester.

## Figures and Tables

**Table 1:** Characteristics of the participants.

	All (n = 825)		No and Mild (n = 682)		Moderate and Severe (n = 143)		*p*
	Median (Q1–Q3) or n (%)		Median (Q1–Q3) or n (%)		Median (Q1–Q3) or n (%)		
Gestational week while answering the questionnaire	22.0	(19.0–24.0)	22.0	(19.0–24.0)	20.0	(18.0–23.0)	<0.001 ^a^
Age (n = 790)	34.0	(31.0–38.0)	34.0	(31.0–38.0)	34.0	(31.0–38.0)	0.959 ^a^
Parity							
Primipara	432	(52.4)	352	(51.6)	80	(55.9)	0.358 ^b^
Multipara	393	(47.6)	330	(48.4)	63	(44.1)	
Number of fetuses							
Singleton	797	(96.6)	656	(96.2)	141	(98.6)	0.204 ^b^
Multiple	28	(3.4)	26	(3.8)	2	(1.4)	
Pre-pregnancy BMI, kg/m^2^ (n = 822)							
<18.5	114	(13.9)	97	(14.3)	17	(12.0)	0.150 ^b^
18.5–24.9	601	(73.1)	502	(73.8)	99	(69.7)	
25.0–29.9	73	(8.9)	57	(8.4)	16	(11.3)	
≥30.0	34	(4.1)	24	(3.5)	10	(7.0)	
Marital status							
Married	810	(98.2)	673	(98.7)	137	(95.8)	0.031 ^b^
Not married	15	(1.8)	9	(1.3)	6	(4.2)	
Education							
Junior high/High school	100	(12.1)	81	(11.9)	19	(13.3)	0.468 ^b^
Vocational training school or junior college	207	(25.1)	165	(24.2)	42	(29.4)	
College	445	(53.9)	373	(54.7)	72	(50.3)	
Postgraduate	73	(8.8)	63	(9.2)	10	(7.0)	
Work							
Yes	533	(64.6)	435	(63.8)	98	(68.5)	0.292 ^b^
No	292	(35.4)	247	(36.2)	45	(31.5)	
Household income							
<7 million yen	402	(48.7)	330	(48.4)	72	(50.3)	0.713 ^b^
≥7 million yen	423	(51.3)	352	(51.6)	71	(49.7)	
Smoking during pregnancy							
No	796	(96.5)	654	(95.9)	142	(99.3)	0.045 ^b^
Yes	29	(3.5)	28	(4.1)	1	(0.7)	
City							
Yamagata	159	(19.3)	139	(20.4)	20	(14.0)	0.127 ^b^
Tokyo	292	(35.4)	239	(35.0)	53	(37.1)	
Osaka	223	(27.0)	187	(27.4)	36	(25.2)	
Fukuoka	151	(18.3)	117	(17.2)	34	(23.8)	

^a^ Mann–Whitney U test; ^b^ Chi-square test. Q1 for the 25th quartile and Q3 for the 75th quartile. BMI, body mass index.

**Table 2:** The energy intakes, body-mass-adjusted intakes of macronutrients and energy-adjusted intakes of the micronutrients.

	All (n = 755)	No and Mild (n = 634)	Moderate and Severe (n = 121)	*p*
	Median (Q1–Q3) or n (%)	Median (Q1–Q3) or n (%)	Median (Q1–Q3) or n (%)	
Energy (kcal)	1494.0 (1278.1–1767.3)	1493.2 (1279.7–1765.8)	1501.0 (1250.3–1779.5)	0.902
Protein (g/kg of body mass)	1.0 (0.8–1.2)	1.0 (0.8–1.2)	1.0 (0.8–1.1)	0.573
Magnesium (mg/1000 kcal)	122.6 (107.3–138.9)	122.7 (107.1–139.5)	121.6 (108.2–136.5)	0.944
Iron (mg/1000 kcal)	3.9 (3.4–4.5)	3.8 (3.3–4.5)	3.9 (3.4–4.5)	0.797
Zinc (mg/1000 kcal)	4.4 (4.1–4.8)	4.4 (4.1–4.8)	4.4 (4.0–4.8)	0.607
Copper (mg/1000 kcal)	0.6 (0.5–0.7)	0.6 (0.5–0.7)	0.6 (0.6–0.7)	0.355
Vitamin B1 (mg/1000 kcal)	0.4 (0.4–0.5)	0.4 (0.4–0.5)	0.4 (0.4–0.5)	0.666
Vitamin B2 (mg/1000 kcal)	0.7 (0.6–0.8)	0.7 (0.6–0.8)	0.6 (0.5–0.7)	0.179
Vitamin B6 (mg/1000 kcal)	0.6 (0.5–0.7)	0.6 (0.5–0.7)	0.6 (0.5–0.7)	0.766
Vitamin B12 (mg/1000 kcal)	3.4 (2.5–4.8)	3.4 (2.5–4.7)	3.6 (2.5–5.4)	0.215
Folate (mg/1000 kcal)	154.3 (126.1–189.5)	155.8 (124.9–190.9)	150.9 (127.9–180.7)	0.542
Vitamin C (mg/1000 kcal)	56.5 (41.0–73.7)	56.5 (41.2–73.1)	57.0 (40.4–77.8)	0.635

Mann–Whitney U test was performed. Intakes of energy, protein, magnesium, iron, zinc, copper, vitamin B1, vitamin B2, vitamin B6, vitamin B12, folate, vitamin C were estimated using a brief-type diet history questionnaire (BDHQ). Q1, 25th quartile; Q3, 75th quartile; E, energy.

**Table 3:** The estimated energy-adjusted intakes of the food groups (g/1000 kcal).

	All (n = 755)	No and Mild (n = 634)	Moderate and Severe (n = 121)	*p*
	Median (Q1–Q3)	Median (Q1–Q3)	Median (Q1–Q3)	
Rice	158.7 (104.2–201.3)	160.9 (104.9–202.3)	148.3 (101.1–200.6)	0.372
Bread	19.6 (11.7–33.9)	19.8 (11.6–33.9)	19.3 (12.3–33.6)	0.670
Noodles	38.4 (24.2–59.8)	36.9 (23.7–56.7)	48.6 (28.4–72.9)	<0.001
Bean	28.1 (16.1–43.7)	28.8 (15.9–44.8)	26.7 (16.8–38.8)	0.302
Vegetables	110.6 (80.6–151.9)	111.2 (81.7–154.0)	106.1 (73.9–141.3)	0.133
Pickled vegetables	2.1 (0.0–6.4)	2.0 (0.0–6.0)	3.1 (0.8–7.9)	0.013
Fruit	44.4 (0.0–39.9)	43.9 (0.0–39.4)	45.4 (0.0–42.5)	0.576
Seaweed	3.0 (1.4–7.0)	3.0 (1.4–7.0)	3.1 (1.4–7.0)	0.965
Red meat	20.2 (15.7–26.1)	20.1 (15.5–26.0)	20.8 (16.1–26.2)	0.727
Processed meat	20.2 (15.7–26.1)	20.1 (15.5–26.0)	20.8 (16.1–26.2)	0.727
Egg	17.8 (12.3–28.6)	17.5 (12.3–28.1)	18.9 (12.6–29.9)	0.489
Alcohol	0.0 (0.0–0.0)	0.0 (0.0–0.0)	0.0 (0.0–0.0)	0.620
Tea	14.0 (0.0–56.7)	12.7 (0.0–53.7)	19.3 (0.0–76.4)	0.031
Juice	42.7 (11.4–89.7)	36.9 (10.4–85.4)	65.7 (23.2–122.8)	<0.001
Coffee	6.5 (0.0–39.9)	6.3 (0.0–39.4)	6.8 (0.0–42.5)	0.612

Mann–Whitney U test was performed. Energy-adjusted intakes of rice, bread, noodles, beans, vegetables, pickled vegetables, fruit, seaweed, red meat, processed meat, eggs, alcohol, tea, juice, and coffee were estimated using a brief-type diet history questionnaire (BDHQ). Q1, 25th quartile and Q3, 75th quartile.

**Table 4:** Dietary habits.

	All (n = 825)		No and Mild (n = 682)		Moderate and Severe (n = 143)		*p*
	Median (Q1–Q3) or n (%)		Median (Q1–Q3) or n (%)		Median (Q1–Q3) or n (%)		
Number of meals including snack	4.0	(3.0–4.0)	4.0	(3.0–4.0)	4.0	(3.0–4.0)	0.210 ^a^
Skip breakfast							
Yes	147	(17.8)	110	(16.1)	37	(25.9)	0.008 ^b^
No	678	(82.2)	572	(83.9)	106	(74.1)	
Skipped meal (weekday)							
Yes	199	(24.1)	150	(22.0)	49	(34.3)	0.003 ^b^
No	626	(75.9)	532	(78.0)	94	(65.7)	
Check label: nutrients							
Yes	496	(60.1)	421	(61.7)	75	(52.4)	0.048 ^b^
No	329	(39.9)	261	(38.3)	68	(47.6)	
Check label: calories							
Yes	495	(60.0)	417	(61.1)	78	(54.5)	0.159 ^b^
No	330	(40.0)	265	(38.9)	65	(45.5)	
Prepare meals							
by self	692	(92.1)	575	(91.9)	117	(93.6)	0.589 ^b^
the others	59	(7.9)	51	(8.1)	8	(6.4)	

^a^ Mann–Whitney U test; ^b^ Chi-square test. Information on the number of meals, skipped meals on weekdays, nutritional label checks, and who prepares the meal was obtained from the questionnaire. Q1, 25th quartile; Q3, 75th quartile.

**Table 5:** GWG, birth weight, and delivery week.

	All		No and Mild		Moderate and Severe		*p*
	Median (Q1–Q3) or n (%)		Median (Q1–Q3) or n (%)		Median (Q1–Q3) or n (%)		
Total GWG (n = 684) (kg)	9.9	(7.8–12.0)	10.0	(7.9–12.1)	9.0	(6.4–11.4)	0.007 ^a^
Pre–pregnancy BMI (kg/m^2^)							
<18.5 (n = 94)	10.4	(8.4–12.0)	10.0	(8.7–12.0)	9.8	(7.5–12.0)	0.415 ^a^
18.5–24.9 (n = 500)	10.1	(8.0–12.1)	10.1	(8.1–12.3)	9.6	(7.2–11.4)	0.050 ^a^
25.0–29.9 (n = 58)	7.6	(4.4–11.6)	7.8	(3.2–11.1)	5.8	(3.3–10.1)	0.824 ^a^
≥30.0 (n = 30)	6.7	(2.8–8.7)	7.7	(2.6–10.7)	5.2	(3.5–8.0)	0.689 ^a^
GWG (kg)							
1st trimester (around 12 week, n = 603)	0.3	(−1.0–1.6)	0.5	(−0.8–1.7)	−0.3	(−2.0–0.9)	<0.001 ^a^
Pre-pregnancy BMI (kg/m^2^)							
<18.5 (n = 84)	0.7	(−0.4–1.7)	0.8	(−0.4–1.8)	0.4	(−1.0–1.1)	0.171 ^a^
18.5–24.9 (n = 434)	0.4	(−0.9–1.6)	0.5	(−0.8–1.7)	−0.2	(−1.8–0.9)	0.003 ^a^
25.0–29.9 (n = 53)	−0.3	(−2.8–1.8)	−0.2	(−2.9–2.1)	−0.9	(−3.2–1.0)	0.273 ^a^
≥30.0 (n = 30)	−0.9	(−3.7–0.4)	−0.8	(−4.0–0.4)	−1.1	(−3.1–1.6)	0.722 ^a^
2nd trimester (around 24 week, n = 644)	4.1	(2.1–6.0)	4.1	(2.4–6.0)	3.4	(0.7–5.6)	0.002 ^a^
Pre-pregnancy BMI (kg/m^2^)							
<18.5 (n = 86)	4.6	(2.4–6.4)	4.7	(2.9–6.7)	4.0	(2.1–5.7)	0.387 ^a^
18.5–24.9 (n = 471)	4.3	(2.4–6.0)	4.3	(2.5–6.0)	3.4	(1.0–5.7)	0.008 ^a^
25.0–29.9 (n = 55)	2.8	(0.3–5.2)	2.8	(0.6–5.5)	3.2	(−0.4–5.6)	0.862 ^a^
≥30.0 (n = 30)	0.6	(−1.5–3.8)	0.6	(−1.8–3.6)	0.0	(−1.4–3.8)	0.929 ^a^
Insufficient GWG (n = 654)							
Yes	337	(51.5)	271	(50.5)	66.0	(56.4)	0.262 ^b^
No	317	(48.5)	266	(49.5)	51.0	(43.6)	
GWG increase rate (kg/week)							
Through pregnancy (n = 684)	0.26	(0.20–0.31)	0.26	(0.21–0.32)	0.24	(0.17–0.30)	0.009 ^a^
Pre-pregnancy BMI (kg/m^2^)							
<18.5 (n = 94)	0.27	(0.22–0.32)	0.27	(0.22–0.32)	0.27	(0.20–0.31)	0.509 ^a^
18.5–24.9 (n = 497)	0.26	(0.21–0.31)	0.26	(0.21–0.32)	0.25	(0.19–0.30)	0.050 ^a^
25.0–29.9 (n = 58)	0.20	(0.11–0.30)	0.21	(0.14–0.31)	0.15	(0.08–0.30)	0.817 ^a^
≥30.0 (n = 30)	0.17	(0.07–0.24)	0.19	(0.07–0.27)	0.13	(0.09–0.20)	0.625 ^a^
1^st^ trimester (around 12 week, n = 603)	0.03	(−0.07–0.12)	0.03	(−0.06–0.13)	−0.02	(−0.15–0.08)	<0.001 ^a^
Pre-pregnancy BMI (kg/m^2^)							
<18.5 (n = 84)	0.05	(−0.03–0.12)	0.05	(−0.03–0.13)	0.03	(−0.08–0.07)	0.177 ^a^
18.5–24.9 (n = 434)	0.03	(−0.07–0.12)	0.04	(−0.06–0.13)	−0.01	(−0.13–0.08)	0.005 ^a^
25.0–29.9 (n = 53)	−0.03	(−0.21–0.12)	−0.01	(−0.20–0.14)	−0.08	(−0.26–0.07)	0.230 ^a^
≥30.0 (n = 30)	−0.07	(−0.28–0.03)	−0.06	(−0.32–0.03)	−0.11	(−0.25–0.14)	0.790 ^a^
1st to 2nd trimester (around 12–24 week, n = 566)	0.36	(0.27–0.46)	0.36	(0.27–0.46)	0.36	(0.25–0.45)	0.438 ^a^
Pre-pregnancy BMI (kg/m^2^)							
<18.5 (n = 76)	0.41	(0.29–0.48)	0.41	(0.30–0.49)	0.40	(0.28–0.46)	0.678 ^a^
18.5–24.9 (n = 407)	0.37	(0.28–0.46)	0.36	(0.28–0.46)	0.38	(0.28–0.46)	0.572 ^a^
25.0–29.9 (n = 51)	0.29	(0.20–0.41)	0.28	(0.17–0.42)	0.29	(0.22–0.42)	0.559 ^a^
≥30.0 (n = 30)	0.24	(0.05–0.38)	0.30	(0.09–0.46)	0.20	(0.04–0.25)	0.114 ^a^
Birth weight (n = 743) (g)	3020.0	(2752.0–3288.0)	3024.0	(2751.0–3291.0)	3000.0	(2751.5–3223.0)	0.482 ^c^
Pre-pregnancy BMI (kg/m^2^)							
<18.5 (n = 102)	2950.0	(2669.5–3206.5)	2956.0	(2657.5–3208.5)	2880.0	(2702.5–3086.5)	0.956 ^c^
18.5–24.9 (n = 541)	3024.0	(2766.0–3294.0)	3024.0	(2764.0–3307.0)	3006.0	(2772.0–3199.5)	0.407 ^c^
25.0–29.9 (n = 66)	3112.0	(2792.0–3314.3)	3130.0	(2822.5–3288.0)	3000.0	(2713.0–3339.8)	0.782 ^c^
≥30.0 (n = 32)	3150.0	(2868.0–3381.5)	3146.0	(2797.0–3350.0)	3146.0	(2797.0–3350.0)	0.711 ^c^
Low birth weight <2500 g							
Yes	48	(7.0)	36	(6.5)	12.0	(9.5)	0.246 ^b^
No	635	(93.0)	521	(93.5)	114.0	(90.5)	
Delivery week (n = 736)	39.0	(38.0–40.0)	39.0	(38.0–40.0)	39.0	(37.0–40.0)	0.783 ^a^
Preterm birth <37 w							
Yes	52	(7.1)	44	(7.3)	8.0	(6.0)	0.604 ^b^
No	684	(92.9)	558	(92.7)	126.0	(94.0)	

^a^ Mann–Whitney U test, ^b^ Chi-square test, ^c^ *t*-test; Q1, 25th quartile and Q3, 75th quartile. GWG, Gestational weight gain; BMI, body mass index.

## Data Availability

The data presented in this study are not publicly available. These will be made available upon reasonable request from the corresponding author.
